# Alagille Syndrome and the Liver: Current Insights

**DOI:** 10.5005/jp-journals-10018-1280

**Published:** 2019-02-01

**Authors:** Shivaram P Singh, Girish K Pati

**Affiliations:** 1 Department of Gastroenterology, Sriram Chandra Bhanj Medical College, Cuttack, Odisha, India; 2 Department of Gastroenterology, Institute of Medical Sciences and Sum Hospital, Bhubaneswar, Odisha, India

**Keywords:** Alagille syndrome, Bile duct paucity, Butter fly vertebra cholestasis, Posterior embryotoxon

## Abstract

Alagille syndrome (ALGS) is an autosomal dominant disorder, with multisystem involvement, which usually occurs due to Notch signaling pathway defects, mostly due to *JAG1* mutation (ALGS type 1), but rarely due to neurogenic locus notch homolog protein (NOTCH2) mutation (ALGS type 2). It was suspected in cases having at least three out of five major clinical criteria: cholestasis with a paucity of the bile duct, congenital cardiac defects, ocular posterior embryotoxon, typical facial features, and skeletal malformation. Till date, no early predictive marker for hepatic outcome in ALGS has found. No genotypic or, phenotype features or correlation could predict the development of endstage liver disease, which poses a unique management challenge. Cases with progressive liver damage, unremitting cholestasis and intractable pruritus often depend on liver transplantation as last resort. The cardiac, and renal status should be well accessed before liver transplant for the better post-transplantation outcome. Most of the clinical manifestations usually improve the following transplant, except any change in stature. The post liver transplantation outcome was usually comparable with other conditions which require liver transplantation as a last resort, but in this disease the effect of long term immunosuppression on other affected systems not evaluated well till date. Therefore long term post transplant prospective study is required to address these issues.

**How to cite this article:** Singh SP, Pati GK. Alagille Syndrome and the Liver: Current Insights. Euroasian J Hepatogastroenterol, 2018;8(2):140-147

## BACKGROUND

Alagille syndrome (ALGS) is an autosomal dominant disorder, which may variably involve multiple systems.^1^ One out of 30,000 subjects may have ALGS.^1^ In 1969, Alagille et al. first reported Alagille syndrome.^2^ In 1973, Watson and Miller also reported the similar type of cases.^3^ Alagille et al. once again reported a similar type of cases in 1975.^4^ Therefore it is also known as Alagille-Watson syndrome or arteriohepatic dysplasia due to preferential hepatic and vascular involvement. It is usually caused by genetic mutations. In this disease, most commonly *JAG1* mutation was observed, and very rare mutation of the NOTCH2 locus found. Earlier it was considered as a predominant hepatic disorder with varied multiple system involvement. Subsequently, it was found that cases with ALGS having *JAG1* or NOTCH2 mutations may lack hepatic manifestations.^5,6^ Most of the cases (approximately 97%) suffer from haploinsuf-ficiency of the *JAG1* gene.^7,8^ Most commonly JAGGED1 mutation occurs on 20p11.2-20p12 chromosome, or rarely deletion take place at this putative locus to give rise to ALGS.^7,8^ Very rarely (approximately 1% of cases) NOTCH2 mutation occur in this disease.^3,9^ Both of the genes are components of the NOTCH signaling pathway. In approximately 60% of cases, sporadic mutations (newer mutation in the proband locus) with a higher rate of germline mosaicism commonly occur.^10,11^ More than 430 *JAG1* mutations have been reported in patients with ALGS till date.^10,11^ The salient features of ALGS is the paucity of intrahepatic bile ducts, and presence of at least three out of five main clinical criterias cholestatic liver disease, cardiovascular malformation, defective skeletal system, ocular malformations, and typical facies.^5^ Sometimes ALGS cases with *JAG1* mutation do not present with all the typical characteristic features or, rarely present with atypical clinical manifestations, which suggests that ALGS may be representative of the tip of the iceberg of *JAG1* mutation-related disorders.^12^ It has been postulated that the name ALGS is appropriate for cases having predominant liver-related manifestations, whereas the wider nomenclature, i.e. *JAG1* disease, should be ideally used for all of the carriers of the mutations, lacking hepatic manifestations. Although the management of this multisystem disease is gradually evolving with time, but till date liver transplantation is the only definitive treatment of choice. The principal difficulty in the optimization of liver care is changing the pattern of the natural outcome of liver disease. Although infants and younger children commonly present with cholestatic symptoms, rarely some children may improve of their cholestatic symptoms completely despite of having very little residual liver capacity.^13^ Till date, there are no early clinical, biochemical, or radiological markers which can predict hepatic outcome in ALGS with certainty. Also, there are no correlations between the genotype and phenotype which could certainly predict the development of end-stage liver disease in ALGS. Because of the above mentioned factors, it becomes very difficult to appropriately manage ALGS cases. Above all younger children with cholestasis may have compensated chronic liver disease with better hepatic outcome in later life. The cases having cardiovascular malformations usually have poor long-term prognosis, as they suffer from poor post liver transplant outcome, due to compromised cardiovascular status. It becomes immensely difficult to decide the appropriate timing for liver transplant in ALGS cases because of all these shortcomings. Till date, only 500 cases of ALGS reported all over the World.^14^ In Indian subcontinent only six cases of ALGS reported so far.^15,16^ A recent case report from India highlighted the fact that case with ALGS, may have early onset dermatological manifestations and chronic liver disease (CLD), which is an unusual presentation not described in the literature.^16^

### Clinical Diagnosis and Diagnostic Criteria

The possibility of ALGS should be suspected in cases presenting with neonatal liver disease and conjugated hyperbilirubinemia; indeed, the distinguishing feature of this disease is an early clinical presentation. In ALGS, bile duct paucity (decreased bile duct-to-portal tract ratio: < 0.4) should be essentially present; along with other diagnostic criteria: at least three out of five major clinical features: hepatocellular cholestasis; ocular abnormalities (in particular posterior embryotoxon); typical facial dys-morphism (triangular face, prominent forehead, deeply placed eyes with wide intercanthal distance, pointed chin, and bulbous tipped straight nose); cardiovascular malformation (in particular peripheral pulmonary artery stenosis); and skeletal deformity (In particular butterfly vertebrae). Also, renovascular malformation and malformed vasculature of other organs (In particular head and neck) may be found in cases with ALGS.^6,17,18^ Usually, ALGS was suspected in cases having a paucity of the bile duct (in the liver histology study) along with at least three out of the five above mentioned criteria. In the recent era, the phenotypic criteria of ALGS will revised and modified; which states that at least three major clinical features may be sufficient to reach at a diagnosis. Even presence of at least two major clinical features along with confirmed diagnosis of ALGS in a first-degree relative may be enough for a diagnosis of ALGS. In the current era, Liver biopsy is no longer required to arrive a diagnosis of ALGS, as the clinico-biochemical evidence of cholestasis is adequate enough to satisfy this criterion.

### Systemic Manifestations and Clinical Description

ALGS usually affects multiple systems and may have a variable presentation: may present with severe hepa-tocellular or cardiovascular compromise or; may have indolent presentation such as mildly deranged liver function, abnormal cardiac sound, butterfly vertebrae, posterior embryotoxon or typical facies. It is very difficult to diagnose, because of variable presentation of ALGS.^19^ Even subjects from the same family with a similar type of genetic mutation may have variable presentation.^12^ In a previous study, 47% subjects out of total 53 relatives of affected individuals, who had genetic mutation could not satisfy the classic clinical diagnostic criteria of ALGS; therefore without molecular diagnostic measures, these individuals might not be properly diagnosed; which signifies importance of molecular diagnostic measures in establishing the diagnosis of ALGS.^12^ ALGS cases may have variable disease progression and mortality basing on types and severity of involved organ. Early mortality is usually determined by cardiovascular or hepatocellular disease; whereas late mortality is usually dependant on severity of vascular accidents.^20,21^ Previous case reports suggested that prenatal diagnosis of ALGS was possible by using a dedicated ultrasound scan, which may reveal presence of lower thoracic hemivertebrae, cardiac malformations, or, agenesis of the gallbladder, in fetus, having *JAG1* mutation.^22^ Emerick et al.^20^ and Subramaniam et al.^23^ vividly reviewed different clinical manifestations of ALGS.

### Hepatic Manifestations

Persistent cholestasis usually occurs in most (approximately 95%) cases,^20^ and, these patients may have neonatal or, early infantile (within first 3 months of life) unremitting jaundice. The cases usually present with deranged liver function test (Increased total and direct bilirubin), increased bile acid concentration in the serum, generalized pruritus, xanthomas and rarely retarded growth. Currently, there is a growing consensus that liver biopsy is not mandatory if clinico-biochemical evidence of cholestasis along with other characteristic manifestations of Alagillle syndrome are present. Liver histology typically reveals a reduction in the concentration of the intrahepatic bile ducts (bile duct to portal tract ratio < 0.4), although ductal proliferation and portal inflammation, the characteristic findings of biliary atresia may be rarely found in the newborn cases with Alagille syndrome. It is therefore essential to meticulously distinguish between these two diseases by appropriate investigations, as the patients with biliary atresia may depend on the Kasai procedure for short term survival, which might not be of any help in ALGS cases.^24^ Decrease in the concentration of bile duct compared to the concentration of portal tract seems to be relentlessly progressive with time, and might be more common in cases with late infancy and early childhood presentation compared to early infancy presentation. The paucity of the bile duct is commonly found in 60% of infants aged less than 6 months, whereas it was more common in older infants (commonly found in 95% of infants aged more than 6 months. Persistent cholestasis, and progressive hepatocellular damage leading to cirrhosis and hepatic failure with the progression of time, commonly affect around 15% of cases, who need a liver transplant as a last resort for survival.^20^ Till date, no well validated prognostic marker described in the literature, which could effectively predict which group of children will land in chronic liver failure, and which group of children would have indolent course. Also, no genotypic, histological, or radiological predictive markers have been developed till date which could confidently predict the severity of the liver disease. It has been reported that cases who subsequently landed in progressive liver disease, usually had persistently elevated total and conjugated bilirubin level, and increased serum cholesterol; however, this needs to be validated in future by long term prospective studies.^25^ Few number of patients have no hepatic manifestations, which was never conceivable earlier.^12,26^ Mouzaki et al. reported that increased serum bilirubin during early childhood period (within 1 to 2 years of age), hepatic fibrosis and presence of xanthoma may clinch towards worse hepatic outcomes in future in cases with ALGS; which needs to be well validated by long term prospective studies.^27^

### Cardiovascular Manifestations

Most (90-97%) of the cases with ALGS may have cardiovascular signs ranging from abnormal heart sounds to significant cardiovascular malformations.^20,26^ Pulmonary vasculature is characteristically involved in these cases; with peripheral pulmonic stenosis (PPS) as the most common finding (in 67% cases with ALGS).^20^ Tetralogy of Fallot (TOF) is the most common complex cardiac malformation (in 7 -16% of cases).^20^ The occurrence of other cardiac defects in decreasing order are ventricular septal defect, atrial septal defect, aortic stenosis, and coarctation of the aorta. Congenital cardiac disease may be the sole finding in cases of ALGS with positive genotype. Several generations in the families with *JAG1* mutations may have PPS, despite of any hepatocellular abnormality.^26^ Presence of complex congenital heart disease such as TOF seems to be an important indicator of early mortality, as it is commonly associated with PPS, whereas liver related conditions amount significantly for deaths in later life.

### Ocular Manifestations

Posterior embryotoxon is the most common ocular finding in cases with ALGS. Characteristic prominent Schwalbe’s ring can be diagnosed by slit-lamp examination in this disease. Posterior embryotoxon has been reported in 78 to 89% of individuals with ALGS.^20,28^

It is the defect in the anterior chamber of the eye and does not affect visual acuity. It can be found in 8 to 15% of normal subjects and in approximately 70% of patients, harboring 22q11 locus deletion.^29^ Optic disk drusen is seen in 90% children with ALGS, which can be detected by the ocular ultrasonographic study. The retinal pigmentary change was found in 32% cases with ALGS.^28^ These ocular findings were previously thought to be due to vitamin deficiency, but they can also be found in cases with absolutely normal vitamins A and E concentration in the serum. Nischal et al.^30^ reported the presence of unilateral optic disk drusen in 95% of patients, whereas 80% of cases with hepatic involvement suffer from bilateral optic disc drusen. Despite all these ocular abnormalities no changes in visual acuity found in this syndrome.

### Skeletal Manifestations

Butterfly shaped thoracic vertebrae are the commonest skeletal abnormality. It usually occurs due to the malformed cleft formation in the vertebral bodies. Approximately 33 to 93% of cases usually have butterfly vertebrae.^21,31,32^ Square shaped proximal part of fingers with tapered distal phalanges and extra digital flexion creases were other skeletal abnormalities.^33^ Due to cholestasis and/or defective formation of bones, there is a very high chance of long bone pathological fractures.^34^A retrospective study reported that 28% of cases with mean age of 5 years suffered from the increased occurrence of pathological fracture mostly in the lower limb (70% of cases) in the families with ALGS.^34^

### Facial Features

Children with ALGS may present with typical facial dysmorphism. These abnormal facial features constitute prominent forehead, deeply and widely placed eyes (Hypertelorism), upward slanting of palpebral fissures, flat nasal bridge, bulbous tipped nose, prominent and large ears, prominent mandible (prognathism), and a pointed chin giving rise to triangular facial appearance.^35^ With the progress of time gradually the triangular shape of face become less marked, whereas prognathism becomes well marked. The ALGS cases with *JAG1* mutations usually present with this type of facial dysmorphism,^1,36^ whereas cases with NOTCH2 mutation usually do not have facial dysmorphism.

### Renal Abnormalities

Cases with ALGS may have small, hyperechoic kidneys, renal cysts, and obstructive uropathy features.^20^ Children with ALGS may present with renal tubular acidosis in approx 74% of cases.^20^ Renal abnormalities might be more common in cases having NOTCH2 mutations compared to JAG mutation.^9^ Renal artery stenosis and renovascular hypertension may be found in adults with ALGS.^37^

### Vascular Events

Vascular accidents may occur in of approximately 15% of cases,^20^ 34% cases died because vascular accidents in one series.^21^ Relatively minor head trauma may lead to devastating intracranial bleeding. Previous reports found vascular anomalies affecting middle cerebral arteries, basilar artery, carotid artery, and renal artery and may present with the middle aortic syndrome, and Moyamoya syndrome.^21,38^

### Growth

Previous studies reported that ALGS cases may have significant growth retardation.^20,39^ Malnutrition and malabsorption might be responsible for growth retardation, but there might be also other non-nutritional putative factors.^40^ Hypothyroidism may be amongst putative factors. Children may have growth hormone resistance.^41^ Short stature may be because of cholestasis, cardiovascular abnormality, and/or defective bone (50 to 90%).^21,39^ The short stature may also occur as a result of immunodeficiency and recurrent infections in the affected cases.^42^

### Pancreas

Approximately 40% of patients with ALGS may have pancreatic insufficiency.^20,43^ Severity of steatorrhoea is not a good marker of pancreatic insufficiency in this disease as steatorrhoea can occur because of bile duct paucity in this disease. Pancreatic enzyme replacement therapy has been proven to be beneficial. Few patients may suffer from diabetes mellitus in the long run.

### Learning Difficulties

Previous studies noted that a large number of ALGS cases suffered from mental retardation,^44^ which might have had an ascertainment bias. Sixteen percent of cases may have a motor delay. The cases with significant cytogenetic deletions in chromosome 20p12 usually suffered from learning difficulties more frequently compared to cases who did not have this.

### Pregnancy and ALGS

- With the progression of pregnancy, the hepatocellular dysfunction and portal hypertension might worse gradually.
- Similarly, cardiac dysfunction and pulmonary hypertension may be more compromised with a progression of pregnancy
- The fetus may inherit the maternal or paternal ALGS mutation in approximately 50% of cases. Due to intrafamilial variability the severity of clinical manifestations cannot be properly predicted. Therefore, meticulous genetic counseling is required before to conception to avoid any untoward consequences.^45^

## PATHOGENESIS

Bile duct paucity is the sine qua none for the diagnosis of ALGS, and also commonly (80-90%) found in large series of patients till date.^20,46^ Bile duct paucity appears to progress relentlessly with time, leading to gradual deterioration of liver function. All the time, this type of progression might not occur. No putative factors for the causation of bile duct paucity could be well identified till date. The importance of *JAG1* protein in the bile duct development during infancy was not clear till date. Decrease in the number of portal tracts has been reported in this disease.^47^ Few infants with ALGS also suffered from ductular proliferation. Why all these differences happen was not clear till date. The wide variability in the phenotypical presentation in the cases with ALGS raises the questions regarding the primary role of the genotype in the development of clinical presentation, and whether some manifestations could be because of medical complications. Besides, there are ongoing debate on the etiology of the distinctive facies: whether it is a primary malformation, or, it results because of chronic cholestasis.^48^ However, ALGS may readily be distinguished from other forms of the cholestatic disorder,^49^ which strongly suggests that the genotype might play a primary role in the variable clinical presentation. Also, it was hypothesized that ALGS might be a primary vasculopathy disorder. In ALGS, a large number of vascular anomalies can occur,^21^ which suggests that defective angiogenesis might play a pivotal role in the development of the varied clinical presentation. Also, it was proven that mature tubular bile duct formation follows the intrahepatic arterial network development.^50^ It was hypothesized that the genetic mutations in the NOTCH signaling pathway involving either *JAG1* or, NOTCH2 genetic locus might play a major role in angiogenesis and clinical presentation.

### Genetics of ALGS

Usually two genes of the notch signaling pathway are responsible for causation of ALGS. These genes are *JAG1* and NOTCH2. Most (98%) of the cases have mutations in the *JAG1* locus, whereas the rest (2%) cases have mutations in NOTCH2 locus.

### JAG1 (Chromosome 20p12.2)

*JAG1,* a ligand for one of four NOTCH transmembrane receptors, is a cell surface protein, and functions as a key signaling molecule in the NOTCH signaling pathway, which plays a crucial role in development. Approximately 500 genetic mutations have been found to be associated with ALGS so far. Out of these, around 69% of cases had protein-truncating variants (frame-shift and nonsense) genetic mutations.^51,52^

### NOTCH2 (Chromosome 1p12-p11)

NOTCH2 is a member of the NOTCH family of transmembrane receptors. The Notch family members play a crucial role in different evolutionary developmental processes by regulating cell fate decisions. Different Notch receptors (NOTCH1, NOTCH2, NOTCH3, and NOTCH4 in humans) usually share morphological characteristics. These include extracellular domains consisting of multiple epidermal growth factor-like repeats and also different types of intracellular domains. Twelve genetic variants have been identified in eleven unrelated families with ALGS. These were one splice site alteration, one frameshift variant, one nonsense variant, and seven missense variants.^9,53^

### 20p12.2 Microdeletion

The ALGS cases with delayed milestone development and/or deafness should point towards chromosome 20p12.2 interstitial microdeletion, which encompasses the *JAG1* gene (the ALGS critical region). Kamath et al. reported that 21 patients had deletions of the short arm of chromosome 20; whereas eleven patients with normal development had deletions between 95 kb and 4 Mb. The proximal and distal extents of these eleven deletions constitute a 5.4 Mb region, which is the *JAG1*-associated critical region. Rest ten patients had larger deletions between 3.28 Mb and 14.62 Mb, extending outside the critical region, and, characteristically, all of these patients had developmental delay.^54^

### Genotype-phenotype Correlations

Till date no genotype and phenotype clinical correlations reported in the literature.^11,55^ However, two families with *JAG1* missense mutations had cardiac disease despite normal liver.^56,57^ Cases with NOTCH2 mutations usually had an increased number of renal abnormalities.^9^ However, the study sample was too small to draw any consensus statement at present. Basing on the genetic data management plan cannot be properly planned as there is no genotype-phenotype correlations described so far in the literature. Few cases satisfying the classic criteria of ALGS might not have any genetic mutation or deletion involving *JAG1* or NOTCH2 locus, illustrating further genetic heterogeneity. Native Canadian family members had some features of ALGS, such as the presence of bile duct paucity, cholestasis, pulmonary stenosis, and autosomal recessive inheritance despite lack of genetic mutations in the *JAG1* or NOTCH2 genetic locus.^58^

### Diagnosis

ALGS cases can be accessed by their genotype and pheno-type despite significant clinical challenges. For instance, bile ducts paucity, a characteristic finding of ALGS, may be found in so many conditions, such as congenital infections, down syndrome, cystic fibrosis, alpha-1-antitrypsin deficiency, and Zellweger and Ivemark syndromes. Bile duct paucity is not a constant feature in any of the above mentioned disorder. In the current era, any clinical or, biochemical evidence of cholestasis is sufficient enough in clinching the diagnosis, without the need of regular liver biopsy for histological diagnosis in every suspected case. Similarly, pulmonary stenosis and other associated congenital cardiac disorders may occur in isolation or, may present like syndromes; for instance, 22q11 deletion syndrome; but PPS is usually much more specific for ALGS. Hepatic manifestations and PPS are very much prevalent in ALGS cases but might present variably. Kamath et al.^12^ evaluated 53 mutation positive relatives of cases with ALGS. Only 21% of cases had clinical features suggestive of a diagnosis of ALGS; whereas 32% cases had some features of ALGS and 47% of cases did not satisfy the diagnosis of ALGS. In a previous study, out of total 241 cases, *JAG1* mutations were found in 59/135 (44%) probands and 24/106 (23%) of their relatives.^19^ Genetic mutations were reported in 54% of cases in proband study having three features of ALGS, whereas mutations were less common (in 34% cases) found in cases having one or two clinical features of ALGS. This report was based on the accuracy of the data provided by the referring clinicians, but above all, the implications were as follows:

- The *JAG1* mutation pick-up rate was not very high in cases having three features of ALGS;
- Surprisingly the mutation-positive pick-up rate was too high in cases having fewer features of ALGS;
- Treating physicians ought to have a high index of suspicion for ALGS despite lower threshold for testing.

Because of these issues, Tsai^59^ revised the diagnostic criteria for ALGS after the availability of molecular diagnostic testing. In a large cohort study, Warthen et al.^60^ reported *JAG1* mutation in 94% clinically well-defined cases. The pick-up rate may be significantly lower during routine practice. Previous reports suggested that *JAG1* mutation might be absent in approximately 40% of suspected cases of ALGS.^6,61-63^ In these studies the pick-up rate might depend too much on the strictness of the clinical criteria used for testing threshold in probands.

### Management

The cases with ALGS should be evaluated and accessed in a systematic manner. Detailed assessment should be done by treating pediatrician or, physician or, gastroenterolo-gist or by all of them and evaluated by doing regular liver function tests, serum lipid profile, coagulation profile, ultrasound abdomen and pelvis, Scintiscan, and liver biopsy in selected cases; detailed cardiac assessment should be done from time to time; regular ophthalmic assessment; renal color Dopplor ultrasound, renal function tests and vertebral X-ray. In infants and children, regular growth monitoring, psychosexual development, dietary pattern, and nutritional status, renal tubular function, and pancreatic function should be accessed regularly. A multidisciplinary approach is required for this. Supplementary feeding may be necessary in cases having severe malnutrition. Cases with ALGS classically presents with intractable pruritus. The management is usually supportive and may be treated with a choleretic agent like ursodeoxycholic acid, or, other medications such as cholestyramine, colesevelam, rifampin, ondansetron, and naltrexone. Surgical biliary diversion procedures (partial internal biliary diversion and ileal exclusion) have also been tried in ALGS to get rid of intractable pruritus and disfiguring xanthomas.^64,65^ Kasai procedure or, hepatic portoenterostomy was not recommended as it may worsen the natural outcome in ALGS cases.^66^ The ALGS cases usually responds to liver transplantation in a dramatic way. The previous study reported that liver transplantation in ALGS cases resulted in improvement of the liver parameters and some catch-up growth in almost 90% of cases and approximately 80% cases survived for more than 5-year.^67^ Study by Kamath et al. reported lower 1-year survival rate in cases with ALGS compared to biliary atresia (87% *vs.* 96% respectively). This reduced survival in ALGS cases was because of more frequent systemic and vascular involvement in ALGS cases compared to biliary atresia cases.^68^ The fate of liver transplantation usually depends on associated cardiac and renal impairment; therefore both of these systems should be properly evaluated and regularly accessed during pre and post liver transplant period.^69^ Living related donors should undergo molecular genetic testing to rule out the possibility of genetic mutation before to liver transplant. Contact sports should be avoided if splenomegaly is present, as there is a chance of traumatic rupture of the enlarged spleen. Drinking of alcohol should be discouraged as there is high chance of malfunction of the liver.

## CONCLUSION

Alagille syndrome (ALGS) is usually a cholestatic liver disorder affecting multiple systems such as heart, eyes, face, kidneys, central nervous system and skeletal system. It is usually inherited in autosomal dominant faction and caused by mutations in one of the two components of NOTCH signaling pathways such as *JAG1* and NOTCH2 genetic locus. There are no early clinical, bichemical, or radiological predictive markers for assessing hepatic outcome in ALGS. Besides, genotype-phenotype correlations too cannot predict the outcome or development of end-stage liver disease in these patients, posing a unique challenge in the management of ALGS. In view of the complexity in presentation and multi-organ involvement, management of cases with Alagille syndrome should be done by the combined effort of a multidisciplinary team, comprising of medical geneticists, gastroenterologists, hepa-tologists, nutritionist, cardiologists, ophthalmologists, nephrologists, liver transplantation, and if necessary a neurosurgeon for better long term outcome.
